# Systemic lupus Erythematosus and geomagnetic disturbances: a time series analysis

**DOI:** 10.1186/s12940-021-00692-4

**Published:** 2021-03-16

**Authors:** George Stojan, Flavia Giammarino, Michelle Petri

**Affiliations:** 1 Division of Rheumatology, Johns Hopkins University School of Medicine, 1830 East Monument Street Suite 7500, Baltimore, MD 21205 USA; 2 Independent Researcher, London, UK

**Keywords:** Systemic lupus erythematosus, Lupus, Environment, Geomagnetic disturbance, Time series, Disease activity

## Abstract

**Background:**

To examine the influence of solar cycle and geomagnetic effects on SLE disease activity.

**Methods:**

The data used for the analysis consisted of 327 observations of 27-day Physician Global Assessment (PGA) averages from January 1996 to February 2020. The considered geomagnetic indices were the AP index (a daily average level for geomagnetic activity), sunspot number index R (measure of the area of solar surface covered by spots), the F10.7 index (measure of the noise level generated by the sun at a wavelength of 10.7 cm at the earth’s orbit), the AU index (upper auroral electrojet index), and high energy (> 60 Mev) proton flux events. Geomagnetic data were obtained from the Goddard Space Flight Center Space Physics Data Facility. A time series decomposition of the PGA averages was performed as the first step. The linear relationships between the PGA and the geomagnetic indices were examined using parametric statistical methods such as Pearson correlation and linear regression, while the nonlinear relationships were examined using nonparametric statistical methods such as Spearman’s rho and Kernel regression.

**Results:**

After time series deconstruction of PGA averages, the seasonality explained a significant fraction of the variance of the time series (*R*^2^ = 38.7%) with one cycle completed every 16 years. The analysis of the short-term (27-day) relationships indicated that increases in geomagnetic activity Ap index (*p* < 0.1) and high energy proton fluxes (> 60 Mev) (*p* < 0.05) were associated with decreases in SLE disease activity, while increases in the sunspot number index R anticipated decreases in the SLE disease activity expressed as PGA (*p <* 0.05). The short-term correlations became statistically insignificant after adjusting for multiple comparisons using Bonferroni correction. The analysis of the long-term (297 day) relationships indicated stronger negative association between changes in the PGA and changes in the sunspot number index R (*p* < 0.01), AP index (*p <* 0.01), and the F10.7 index (*p <* 0.01). The long-term correlations remained statistically significant after adjusting for multiple comparisons using Bonferroni correction.

**Conclusion:**

The seasonality of the PGA averages (one cycle every 16 years) explains a significant fraction of the variance of the time series. Geomagnetic disturbances, including the level of geomagnetic activity, sunspot numbers, and high proton flux events may influence SLE disease activity. Studies of other geographic locales are needed to validate these findings.

**Supplementary Information:**

The online version contains supplementary material available at 10.1186/s12940-021-00692-4.

## Introduction

The Earth is immersed in a geomagnetic field (GMF) that deflects the flowing ionized particles of solar winds. In doing so, the GMF is impressed and altered by the solar winds [1]. These alterations in GMF are called geomagnetic disturbances. The GMF has different strength and direction in different areas of the Earth. Its magnitudes vary from ~ 35,000 nano Tesla (nT) at the equator to ~ 70,000 nT at the magnetic poles [2]. Several *periodic variations* have also been shown in GMF, mostly related to various solar cycles (especially the 11 year cycle), and others related to Earth’s rotation and situation in its orbit [2].

The idea that variations in solar activity can influence biological phenomena originated with the work of Alexander Chizhevsky in the early twentieth century. He carried out a systematic study of the influence of electric, magnetic and electromagnetic perturbations in the external physicochemical environment on biological, social, and historical events [3]. The advent of space-based observatories such as the joint USA/European Solar and Heliospheric Observatory during the late twentieth century and early twenty-first century facilitated further research into the effect of variations in the sun and earth’s magnetic field on human health. High solar activity associated with excess death from myocardial infarction [4]. Geomagnetic disturbances were directly correlated with erythrocyte aggregation studies and blood velocity of patients with acute myocardial infarction [5] and with blood pressure variability [6]. A strong non-linear correlation between sudden infant death syndrome and geomagnetic activity was described in Ontario, Canada [7]. Geomagnetic disturbances have been postulated to explain the relapsing-remitting nature, chronobiology, and latitudinal prevalence of multiple sclerosis [8]. Giant cell arteritis and rheumatoid arthritis incidence rates were shown to be highly correlated with the AL index, a proxy of the westward auroral electrojet and a measure of geomagnetic activity [9].

We previously described significant seasonal variation in SLE disease activity with more arthritis activity in the spring and summer months, and an increase in renal activity in winter months, significantly higher anti-dsDNA antibody titers in the fall, and a significant variation of global disease activity as measured by SELENA-SLEDAI through the year [10]. We also performed a spatial-time cluster analysis of the Hopkins Lupus Cohort and detected space-time organ specific lupus flare clusters which had multi-year cluster patterns that did not conform to any known pattern of infectious disease or environmental exposure [11].

The only previous study assessing the effect of geomagnetic disturbances in SLE analyzed the fluctuation of antiphospholipid antibodies over time and found that the anti-beta 2 glycoprotein (*p* < 0.0001) and lupus anticoagulant (*p <* 0.05) were more commonly detected on days of major geomagnetic storms [12].

We thus hypothesized that geomagnetic disturbances influence SLE disease activity.

## Methods

### Patients and activity indices

As previously described [13], the Hopkins Lupus Cohort is a prospective cohort study of predictors of lupus flare, atherosclerosis, and health status in SLE. The study cohort includes all patients at the Hopkins Lupus Center who have a clinical diagnosis of SLE and have given informed consent to participate in the study. Subjects enrolled in the cohort are followed quarterly or more frequently if clinically necessary. The clinical features, laboratory testing, and damage accrual data are recorded at the time of entry into the cohort and are updated at each subsequent visit. The Hopkins Lupus Cohort has been approved by the Johns Hopkins University School of Medicine Institutional Review Board and complies with the Health Insurance Portability and Accountability Act. All patients gave written informed consent.

Ninety-five percent of patients fulfilled 4 or more of the American College of Rheumatology (ACR) 1982 revised classification criteria for SLE [14, 15] and/or the SLICC classification criteria for SLE [16]. Disease activity was measured with the Physician Global Assessment (PGA) [17]. The data used for the analysis consisted of 327 observations of 27-day PGA averages from 24 January 1996 to 29 February 2020. A 27-day PGA average was used for analysis due to the specific periodicity of the OMNI dataset [18].

The OMNI data were obtained from the Goddard Space Flight Center/Spatial Physics Data Facility OMNIWeb interface at https://omniweb.gsfc.nasa.gov. OMNI is an open-source 20-spacecraft compilation of near-Earth, solar wind magnetic field and plasma (proton and, since 1971, alpha particle) data, energetic proton fluxes, and geomagnetic activity indices (Kp, Dst, AE) and sunspot number. We used the Ap index, sunspot number index R, the F10.7 index, the AU index, and high energy (> 60 Mev) proton flux events for our analyses. Detailed descriptions of these geomagnetic indices can be located in supplementary materials.

### Statistical methodologies

As a first step we studied the components of the 27-day PGA average time series, specifically its trend, seasonality and noise. The trend was assumed to be a linear function of time, and its parameters were estimated by Ordinary Least Squares (OLS). The seasonality was assumed to be a sinusoidal function of time, and its parameters were also estimated by OLS. The behavior of the noise was consistent with an autoregressive moving average (ARMA) process, and its parameters were obtained by Maximum Likelihood Estimation (MLE).

As a second step we investigated the relationship between changes in the 27-day average PGA and changes in the 27-day averages of the considered geomagnetic indices. We examined both the contemporaneous relationships (specifically, the relationship between changes in the PGA and changes in the geomagnetic indices over the same 27-day period) and the lead-lag relationships (specifically, the relationship between changes in the PGA over a given 27-day period and changes in the geomagnetic indices over the previous 27-day period). We used Pearson’s linear correlation coefficient and univariate linear regression to investigate the presence of linear relationships, while we use Spearman’s rank correlation coefficient and univariate Kernel regression to investigate the presence of nonlinear relationships.

## Results

### Physician global assessment (PGA) time series decomposition

**Fig. 1:**
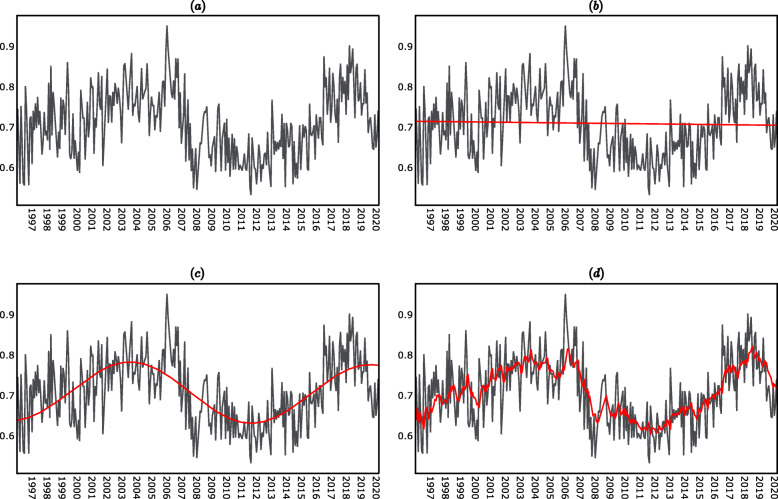
27-day PGA average time series decomposition. **a** 27-day PGA average. **b** 27-day PGA average and fitted trend component. **c** 27-day PGA average and fitted trend and seasonality components. **d** 27-day PGA average and fitted trend, seasonality, and noise components

The plot of the 27-day PGA average time series is shown in panel (a) of Fig. 1.

*Trend.* In what follows *y*_*t*_ denoted the value of the 27-day PGA average on period *t*, with *t* = 1, 2, …, 327. For estimating the trend, *y*_*t*_ was assumed to be described by a linear function of time as in Eq. (1).

1 $$ {y}_t={\beta}_0+{\beta}_1t+{\eta}_t\kern1.5em {\eta}_t\sim \mathcal{N}\left(0,{\sigma}^2\right) $$

The parameters in Eq. (1) were estimated by Ordinary Least Squares (OLS). The regression results were reported in panel (a) of Table 1. The estimated intercept (${\hat{\beta}}_0$) is in line with the sample mean of the time series and was statistically significantly different from zero. The estimated slope (${\hat{\beta}}_1$) was instead not statistically significantly different from zero. The *R*^2^ of the regression was 0.1%. Overall, the regression results indicated that the trend explained only a negligible fraction of the variance of the time series, and therefore the trend was not a significant component of the time series.

**Table 1:** 27-day PGA average time series parameter estimation

***(a) Estimation of trend parameters.***				
*Parameter*	*Estimated Coefficient*	*Standard Error*	*t-statistic* *(value)*	*t-statistic* *(p-value)*
${\hat{\beta}}_0$	0.715	0.009	76.663	0.000
${\hat{\beta}}_1$	−0.00003	0.00005	−0.607	0.544
*Jarque-Bera (p-value)*		0.041	*Log-Likelihood*	344.97
*Durbin-Watson (value)*		0.868	*AIC*	− 685.9
*R-squared*		0.001	*F-statistic (value)*	0.3682
*Adjusted R-squared*		−0.002	*F-statistic (p-value)*	0.5440
***(b) Estimation of seasonality parameters.***				
*Parameter*	*Estimated Coefficient*	*Standard Error*	*t-statistic* *(value)*	*t-statistic* *(p-value)*
$\hat{C}$	−0.0030	0.004	−0.774	0.440
$\hat{A}$	−0.0732	0.005	−14.163	0.000
$\hat{B}$	0.0114	0.005	2.099	0.037
*Jarque-Bera (p-value)*		0.282	*Log-Likelihood*	425.05
*Durbin-Watson (value)*		1.417	*AIC*	− 844.1
*R-squared*		0.387	*F-statistic (value)*	102.4
*Adjusted R-squared*		0.383	*F-statistic (p-value)*	0.000
***(c) Estimation of ARMA parameters.***				
*Parameter*	*Estimated Coefficient*	*Standard* *Error*	*t-statistic* *(value)*	*t-statistic* *(p-value)*
${\hat{\psi}}_1$	0.8705	0.067	13.048	0.000
${\hat{\lambda}}_1$	−0.6805	0.095	−7.133	0.000
*Jarque-Bera (p-value)*		0.36	*Log-Likelihood*	447.152
*Ljung-Box (p-value)*		0.41	*AIC*	− 888.303

In what follows the estimated trend was denoted by

2 $$ {\hat{T}}_t={\hat{\beta}}_0+{\hat{\beta}}_1t $$

The plot of the estimated trend is shown in panel (b) of Fig. 1. The Root Mean Square Error (RMSE) obtained when predicting the time series (i.e. *y*_*t*_) using only the estimated trend component (i.e. ${\hat{T}}_t$) was 0.084. This indicates that, when using only the estimated trend to predict the lupus disease activity, the prediction error was 0.084 on average.

*Seasonality.* In what follows *s*_*t*_ denoted the value of the seasonality function on period *t*, with *t* = 1, 2, …, 327. The seasonality function *s*_*t*_ was assumed to be a sinusoidal function of time as in Eq. (3).

3 $$ {s}_t={\theta}_0+{\theta}_1\cos \left(2\pi \left(\omega t+\phi \right)\right) $$

The interpretation of the parameters in Eq. (3) was the following:

- *θ*_0_ was the mean, which represented the level around which the seasonality function fluctuates.
- *θ*_1_ was the amplitude, which represented the width of the fluctuations of the seasonality function.
- *ω* was the seasonal frequency, which represented the number of cycles of the seasonality function over a given seasonal period.
- *ϕ* was the phase, which defined the time when the seasonality function attained its peak.

The seasonality function in Eq. (3) can be reparametrized as follows

$$ {s}_t={\theta}_0+{\theta}_1\cos \left(2\pi \omega t+2\pi \phi \right) $$

$$ ={\theta}_0+{\theta}_1\left[\cos \left(2\pi \omega t\right)\cos \left(2\pi \phi \right)-\sin \left(2\pi \omega t\right)\sin \left(2\pi \phi \right)\right] $$

$$ ={\theta}_0+\left[{\theta}_1\cos \left(2\pi \phi \right)\right]\cos \left(2\pi \omega t\right)+\left[-{\theta}_1\sin \left(2\pi \phi \right)\right]\sin \left(2\pi \omega t\right) $$

$$ =C+A{x}_{1,t}+B{x}_{2,t} $$

where *C* = *θ*_0_, *A* = *θ*_1_ cos(2*πϕ*), *B* =  − *θ*_1_ sin(2*πϕ*), *x*_1, *t*_ = cos(2*πωt*) and *x*_2, *t*_ = sin(2*πωt*). The parameters *A*, *B* and *C* could be estimated by OLS by fitting a linear regression model to the detrended time series (i.e. ${y}_t-{\hat{T}}_t$) as in Eq. (4).

4 $$ \left({y}_t-{\hat{T}}_t\right)=C+A{x}_{1,t}+B{x}_{2,t}+{\epsilon}_t\kern1.5em {\epsilon}_t\sim \mathcal{N}\left(0,{\gamma}^2\right) $$

The regression results are reported in panel (b) of Table 1. The estimated intercept $\hat{C}$ was not statistically significantly different from zero, which was expected since the detrended time series has zero mean. The estimated coefficients of the sinusoids $\hat{A}$ and $\hat{B}$ were instead both statistically significantly different from zero. The *R*^2^ of the regression is 38.7%. Overall, the regression results indicated that, differently from the trend, the seasonality explained a significant fraction of the variance of the time series.

The estimates of *θ*_0_, *θ*_1_ and *ϕ* were derived from the estimates of *A*, *B* and *C* as follows [19]

$$ {\theta}_0=\hat{C} $$

$$ {\theta}_1=\sqrt{{\hat{A}}^2+{\hat{B}}^2} $$

$$ \hat{\phi}=\left\{\begin{array}{c}\begin{array}{cc}\frac{1}{2\pi }{\tan}^{-1}\left(-\frac{\hat{B}}{\hat{A}}\right)&\ \hat{A}>0\\ {}\frac{1}{2\pi}\left({\tan}^{-1}\left(-\frac{\hat{B}}{\hat{A}}\right)-\pi \right)& \kern0.5em \hat{A}<0,\hat{B}>0\end{array}\\ {}\begin{array}{cc}\frac{1}{2\pi}\left({\tan}^{-1}\left(-\frac{\hat{B}}{\hat{A}}\right)+\pi \right)& \hat{A}<0,\hat{B}\le 0\\ {}-\frac{1}{4}& \hat{A}=0,\hat{B}>0\end{array}\\ {}\begin{array}{cc}\kern0.5em +\frac{1}{4}&\ \hat{A}=0,\hat{B}<0\\ {}\kern10.5em & \kern0.5em \end{array}\end{array}\right. $$

The estimated mean *θ*_0_ was − 0.003, the estimated amplitude *θ*_1_ was 0.074, and the estimated phase *ϕ* was − 0.475.

**Fig. 2:**
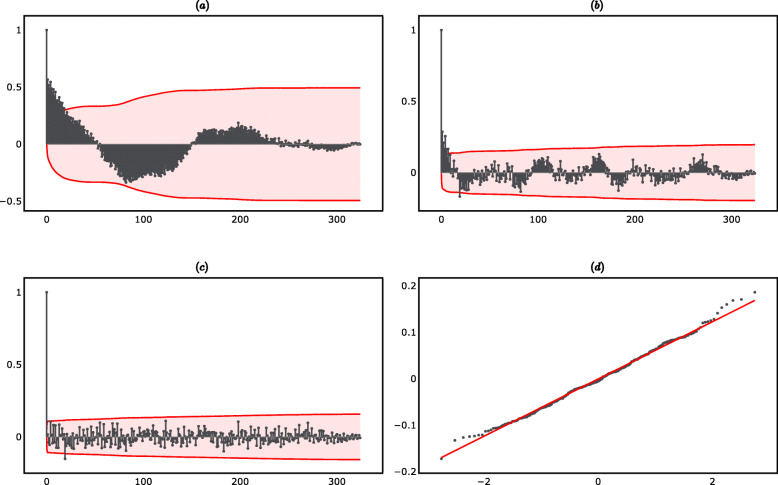
27-day PGA average time series analysis. **a** ACF of detrended 27-day PGA average. **b** ACF of detrended and deseasonalized 27-day PGA average. **c** ACF of ARMA(1,1) residuals. **d** Q-Q plot of ARMA(1,1) residuals

The seasonal frequency *ω* was not estimated directly, but was instead inferred by re-estimating the regression model in Eq. (4) using as input different values of *ω* and by then choosing the value of *ω* which provided the highest *R*^2^. The value of *ω* which maximized the *R*^2^ is 0.062, indicating that the seasonality function completed one cycle every 16.05 years. This is in line with the behavior of the autocorrelation function (ACF) of the detrended time series reported in panel (a) of Fig. 2 (supplementary material), which showed a periodic pattern with a frequency of approximately 220 periods, where the length of each period was 27 days. The most recent seasonal peak was in 2019, and therefore the next seasonal peak is expected in 2035.

In what follows the estimated seasonality function was denoted by

5 $$ {\hat{S}}_t={\hat{\theta}}_0+{\hat{\theta}}_1\cos \left(2\pi \left(\hat{\omega}t+\hat{\phi}\right)\right) $$

The plot of the estimated trend and seasonal components (i.e. ${\hat{T}}_t+{\hat{S}}_t$) is shown in panel (c) of Fig. 1. The RMSE obtained when predicting the time series (i.e. *y*_*t*_) using both the estimated trend component and the estimated seasonal component (i.e. ${\hat{T}}_t+{\hat{S}}_t$) was 0.065. This indicated that, when using both the estimated trend and the estimated seasonality to predict the lupus disease activity, the prediction error was 0.065 on average.

*Noise.* The detrended and deseasonalized time series followed an ARMA (1, 1) process. The ARMA (1, 1) specification was consistent with the plot of the ACF of the detrended and deseasonalized time series shown in panel (b) of Fig. 2 (supplementary material). The appropriateness of the ARMA (1, 1) model choice was confirmed by the results of a grid search, which showed that the ARMA (1, 1) model had the lowest Akaike Information Criterion (AIC) compared to alternative ARMA(p,q) models. The ARMA process was:

6 $$ \left({y}_t-{\hat{T}}_t-{\hat{S}}_{\mathrm{t}}\right)={\psi}_1\left({y}_{t-1}-{\hat{T}}_{t-1}-{\hat{S}}_{t-1}\right)+{\lambda}_1{\kappa}_{t-1}+{\kappa}_t\kern1.5em {\kappa}_t\sim \mathcal{N}\left(0,{\mathcal{X}}^2\right) $$

The parameters of the ARMA (1, 1) model were obtained by Maximum Likelihood Estimation (MLE). The estimation results are reported in panel (c) of Table 1. The estimated autoregressive parameter (${\hat{\psi}}_1$) and moving average parameter (${\hat{\lambda}}_1$) were both statistically significantly different from zero and the behavior of the residuals indicated that the model was correctly specified. In particular, the Ljung-Box test indicated that the residuals were serially uncorrelated, while the Jarque-Bera test indicated that the residuals were normally distributed. The ACF plot and Q-Q plot of the residuals are reported in panels (c) and (d) of Fig. 2 (supplementary material). The plot of the estimated trend, seasonal and noise components is shown in panel (d) of Fig. 1.

### Relationship between physician global assessment (PGA) and geomagnetic indices

The time series of the AU index had 27 missing values, the time series of Proton Flux (> 60 Mev) had 12 missing values, while all other time series had no missing values.

The results of the KPSS test in (supplementary material) indicated that the levels time series (i.e. the time series of the levels of the variables on each period) were non-stationary, while the differences time series (i.e. the time series of the absolute changes of the variables between one period and the next) were stationary, with the exception of the lupus disease activity time series in which case both the levels and the differences were stationary. In view of the results of the KPSS test, the analysis presented in the following sections was conducted using the differences time series instead of the levels time series. The descriptive statistics of the time series are reported in (supplementary material), while the plots of the time series are shown in Figs. 7, 8 and 9 (supplementary material).

#### Short-term linear relationships

Table 2 reports the Pearson’s correlation coefficients between changes in PGA and changes in the geomagnetic indices over 1 period (27 days). The Pearson’s correlation coefficient measures the strength of the linear relationship between two variables. The first column reports the correlations of the changes in PGA with the changes in the geomagnetic indices in the same period (i.e. between the change in PGA over period *t* and the change in each geomagnetic index over the same period *t*), while the second column reports the correlations of the changes in PGA in the current period with the changes in the geomagnetic indices in the previous period (i.e. between the change in PGA over period *t* and the change in each geomagnetic index over the previous period *t* − 1).

**Table 2:** Short-term (27-day) correlations of the changes in PGA with the changes in the geomagnetic indices in the same period (first column) and of the changes in PGA in the current period with the changes in the geomagnetic indices in the previous period (second column)

	Δ*PGA*_*t*_		Δ*PGA*_*t*_
*ΔR*_*t*_	−0.5%	*ΔR*_*t* − 1_	− 10.8%**
*ΔAP*_*t*_	−10.1%*	*ΔAP*_*t* − 1_	1.6%
*ΔF*10.7_*t*_	−1.9%	*ΔF*10.7_*t* − 1_	−6.4%
*ΔAU*_*t*_	−6.4%	*ΔAU*_*t* − 1_	6.3%
*ΔPF*_*t*_	−11.3%**	*ΔPF*_*t* − 1_	5.1%

*: *p*-value <0.1, **: *p*-value < 0.05, ***: *p*-value < 0.01

The results in Table 2 indicate that, over short time periods:

- Increases in R (Sunspot No.) could anticipate decreases in the PGA, i.e. positive *ΔR*_*t* − 1_ tend to be associated with negative Δ*PGA*_*t*_ (*p* < 0.05).
- Increases in the AP index were associated with decreases in the PGA, i.e. positive *ΔAP*_*t*_ tended to be associated with negative Δ*PGA*_*t*_ (*p* < 0.1).
- Increases in the Proton Flux (> 60 Mev) were associated with decreases in the PGA, i.e. positive *ΔPF*_*t*_ tended to be associated with negative Δ*PGA*_*t*_ (*p <* 0.05).

Overall the values of the correlation coefficients reported in Table 2 were relatively small, indicating that the linear relationship between changes in PGA and changes in the geomagnetic indices was in general weak. All the correlations in Table 2 are statistically insignificant after adjusting for multiple comparisons using Bonferroni correction (*p* > 0.1).

**Fig. 3:**
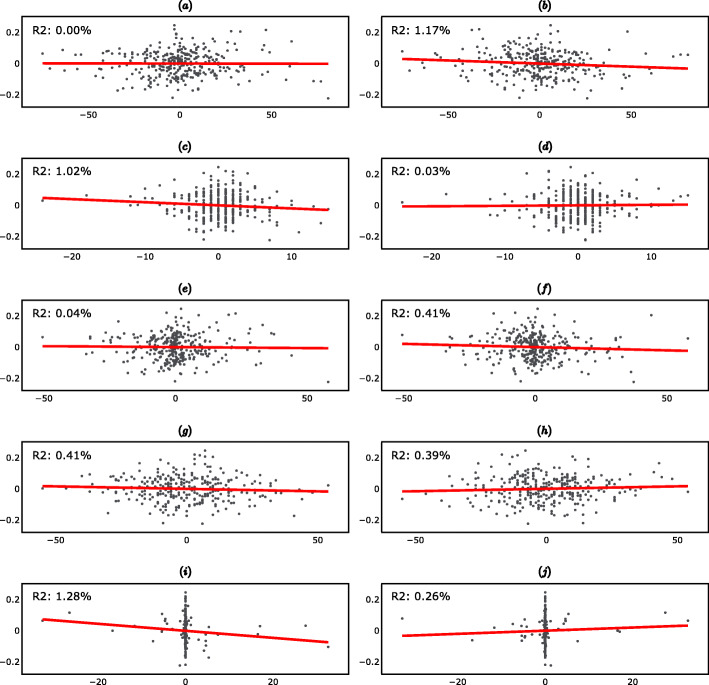
Short-Term Linear Regressions (27-day differences). **a** Scatter plot of ΔPGA_t_ vs ΔR_t_. **b** Scatter plot of ΔPGA_t_ vs ΔR_t-1_. **c** Scatter plot of ΔPGA_t_ vs ΔAP_t_. **d** Scatter plot of ΔPGA_t_ vs ΔAP_t-1_. **e** Scatter plot of ΔPGA_t_ vs ΔF10.7_t._ **f** Scatter plot of ΔPGA_t_vs ΔF10.7_t-1_. **g** Scatter plot of ΔPGA_t_ vs ΔAU_t_. **h** Scatter plot of ΔPGA_t_ vs ΔAU_t-1_. **i** Scatter plot of ΔPGA_t_ vs ΔPF_t_. **j** Scatter plot of ΔPGA_t_ vs ΔPF_t-1_

Figure 3 (supplementary material) shows the fitted linear regressions. Note that, when only one independent variable was included in the regression, the *R*^2^ was equal to the square of the correlation coefficient between the dependent variable and the independent variable, i.e. the *R*^2^ reported in Fig. 3 (supplementary material) are the squares of the correlation coefficients reported in Table 2.

#### Long-term linear relationships

Table 3 reports the Pearson’s correlation coefficients between changes in PGA and changes in the geomagnetic indices over 11 periods (297 days).

**Table 3:** Long-term (297 days) correlations of the changes in PGA with the changes in the geomagnetic indices in the same period (first column) and of the changes in PGA in the current period with the changes in the geomagnetic indices in the previous period (second column)

	*ΔPGA*_*t*_		*ΔPGA*_*t*_
*ΔR*_*t*_	−16.7%***	*ΔR*_*t* − 1_	−16.2%***
*ΔAP*_*t*_	−16.5%***	*ΔAP*_*t* − 1_	−7.9%
*ΔF*10.7_*t*_	− 17.2%***	*ΔF*10.7_*t* − 1_	−13.6%**
*ΔAU*_*t*_	−3.8%	*ΔAU*_*t* − 1_	−1.0%
*ΔPF*_*t*_	−9.4%	*ΔPF*_*t* − 1_	2.9%

*: *p*-value < 0.1, **: *p*-value < 0.05, ***: *p*-value < 0.01

The results in Table 3 indicate that, over long time periods:

- Increases in R (Sunspot No.) are associated to decreases in the PGA, i.e. positive
  *ΔR*_*t*_ are associated with negative Δ*PGA*_*t*_ (*p* < 0.01). Furthermore, increases in R (Sunspot No.) anticipate decreases in the PGA, i.e. positive *ΔR*_*t* − 1_ are associated with negative Δ*PGA*_*t*_ (*p <* 0.01). Both correlations are still statistically significant after adjusting for multiple comparisons using Bonferroni correction (*p* < 0.05).
- Increases in the AP index are associated with decreases in the PGA, i.e. positive ΔAP_t_ are associated with negative Δ*PGA*_*t*_ (*p <* 0.01). The correlation is still statistically significant after adjusting for multiple comparisons using Bonferroni correction (*p <* 0.05).
- Increases in the F10.7 index are associated with decreases in the PGA, i.e. positive *ΔF*10.7_*t*_ are associated with negative Δ*PGA*_*t*_ (*p <* 0.01). Furthermore, increases in the F10.7 index anticipate decreases in the PGA, i.e. positive *ΔF*10.7_*t* − 1_ are associated with negative Δ*PGA*_*t*_ (*p <* 0.05). Both correlations are still statistically significant after adjusting for multiple comparisons using Bonferroni correction (*p <* 0.05 and *p* < 0.1 respectively).

**Fig. 4:**
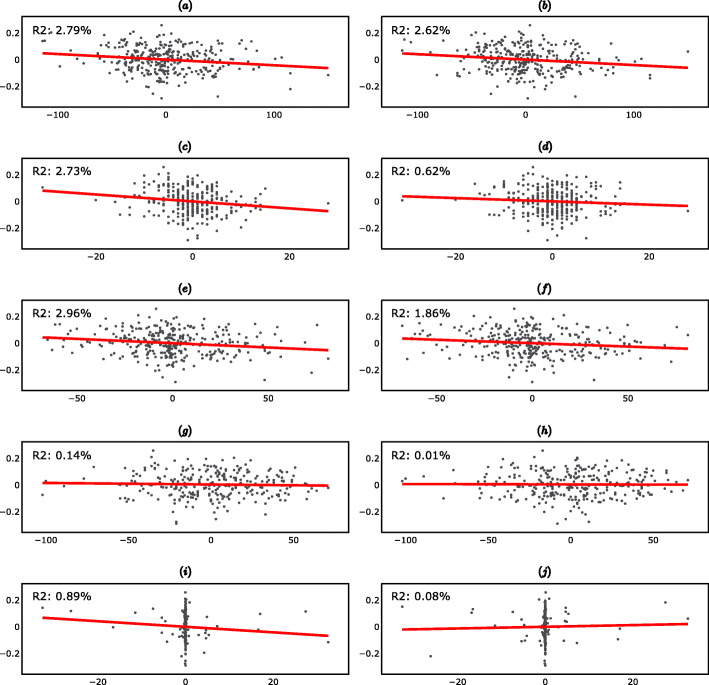
Long-Term Linear Regressions (297 day differences). **a** Scatter plot of ΔPGA_t_ vs ΔR_t_. **b** Scatter plot of ΔPGA_t_ vs ΔR_t-1_. **c** Scatter plot of ΔPGA_t_ vs ΔAP_t_. **d** Scatter plot of ΔPGA_t_ vs ΔAP_t-1_. **e** Scatter plot of ΔPGA_t_ vs ΔF10.7_t_. **f** Scatter plot of ΔPGA_t_ vs ΔF10.7_t-1_. **g** Scatter plot of ΔPGA_t_ vs ΔAU_t_. **h** Scatter plot of ΔPGA_t_ vs ΔAU_t-1_. **i** Scatter plot of ΔPGA_t_ vs ΔPF_t_. **j** Scatter plot of ΔPGA_t_ vs ΔPF_t-1_

The fitted linear regression lines and the corresponding R-squared are reported in Fig. 4 (supplementary material). The interpretation of the R-squared is the same as in Section 2.1.

#### Short-term nonlinear relationships

Table 4 reports the Spearman’s rank correlation coefficients between changes in PGA and changes in the geomagnetic indices over short time periods (27 days). The Spearman’s rank correlation coefficient measures the strength of the monotonic relationship between two variables. The Spearman’s rank correlation coefficient is more general than Pearson’s correlation coefficient, as it also captures nonlinear relationships.

**Table 4:** Short-term (27 days) Spearman’s rank correlation coefficients of changes in PGA with the changes in the geomagnetic indices in the same period (first column) and of the changes in PGA in the current period with the changes in the geomagnetic indices in the previous period (second column)

	*ΔPGA*_*t*_		*ΔPGA*_*t*_
*ΔR*_*t*_	−0.5%	*ΔR*_*t* − 1_	− 11.7%*
*ΔAP*_*t*_	−6.1%	*ΔAP*_*t* − 1_	1.6%
*ΔF*10.7_*t*_	−1.0%	*ΔF*10.7_*t* − 1_	− 8.0%
*ΔAU*_*t*_	− 7.1%	*ΔAU*_*t* − 1_	4.7%
*ΔPF*_*t*_	− 6.1%	*ΔPF*_*t* − 1_	5.8%

*: *p*-value < 0.1, **: *p*-value < 0.05, ***: *p*-value < 0.01

As in the previous section, the first column of Table 4 reports the correlations of the changes in PGA with the changes in the geomagnetic indices in the same period (i.e. between the change in PGA over period *t* and the change in each geomagnetic index over the same period *t*), while the second column reports the correlations of the changes in PGA in the current period with the changes in the geomagnetic indices in the previous period (i.e. between the change in PGA over period *t* and the change in each geomagnetic index over the previous period *t* − 1).

The results in Table 4 indicate that, even after allowing for nonlinear monotonic relationships, over short time periods the association between changes in PGA and changes in the geomagnetic indices was still weak. None of the correlations in Table 4 is statistically significant after adjusting for multiple comparisons using Bonferroni correction (*p* > 0.1).

**Fig. 5:**
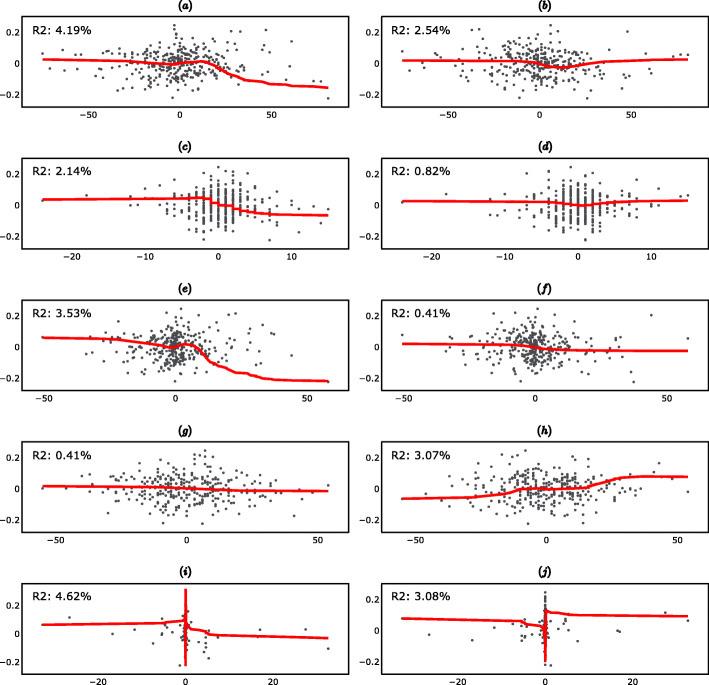
Short-Term Kernel Regressions (27-day differences). **a** Scatter plot of ΔPGA_t_ vs ΔR_t_. **b** Scatter plot of ΔPGA_t_ vs ΔR_t-1_. **c** Scatter plot of ΔPGA_t_ vs ΔAP_t_. **d** Scatter plot of ΔPGA_t_ vs ΔAP_t-1_. **e** Scatter plot of ΔPGA_t_ vs ΔF10.7_t_. **f** Scatter plot of ΔPGA_t_ vs ΔF10.7_t-1_. **g** Scatter plot of ΔPGA_t_ vs ΔAU_t_. **h** Scatter plot of ΔPGA_t_ vs ΔAU_t-1_. **i** Scatter plot of ΔPGA_t_vs ΔPF_t_. **j** Scatter plot of ΔPGA_t_ vs ΔPF_t-1_.

Figure 5 (supplementary section) shows the fitted Kernel regressions. Kernel regression is more general than linear regression as it allows for nonlinear relationships between the dependent variable and the independent variables. Furthermore, in Kernel regression the functional form of the nonlinear relationship is estimated nonparametrically, i.e. it does not need to be specified in advance.

Note that in this case there was no relationship between the *R*^2^ reported in Fig. 5 (supplementary section) and the correlation coefficients reported in Table 4. Note also that, as explained above, Spearman’s rank correlation coefficient measures only monotonic relationships, while as shown in Fig. 5 (supplementary section), Kernel regression also captures non-monotonic relationships.

#### Long-term nonlinear relationships

Table 5 reports the Spearman’s rank correlation coefficients between changes in PGA and changes in the geomagnetic indices over long time periods (297 days).

**Table 5:** Long-term (297 days) Spearman’s rank correlation coefficients of changes in PGA with the changes in the geomagnetic indices in the same period (first column) and of the changes in PGA in the current period with the changes in the geomagnetic indices in the previous period (second column)

	*ΔPGA*_*t*_		*ΔPGA*_*t*_
*ΔR*_*t*_	−11.7%**	*ΔR*_*t* − 1_	− 15.9%***
*ΔAP*_*t*_	−16.7%***	*ΔAP*_*t* − 1_	−6.2%
*ΔF*10.7_*t*_	− 15.2%***	*ΔF*10.7_*t* − 1_	−15.5%***
*ΔAU*_*t*_	−2.7%	*ΔAU*_*t* − 1_	− 0.5%
*ΔPF*_*t*_	− 2.5%	*ΔPF*_*t* − 1_	8.7%

*: *p*-value < 0.1, **: *p*-value < 0.05, ***: p-value < 0.01

The results are similar to the ones reported in Table 3:

- Increases in R (Sunspot No.) are associated with decreases in the PGA, i.e. positive
  *ΔR*_*t*_ are associated with negative (*p* < 0.05). However, this correlation is not statistically significant after adjusting for multiple comparisons using Bonferroni correction (*p* > 0.1). Furthermore, increases in R (Sunspot No.) anticipate decreases in the PGA, i.e. positive *ΔR*_*t* − 1_ tend to be associated with negative ΔPGA_t_ (*p* < 0.01). This correlation is still statistically significant after adjusting for multiple comparisons using Bonferroni correction (*p <* 0.05).
- Increases in the AP index were associated with decreases in the PGA, i.e. positive ΔAP_t_are associated with negative ΔPGA_t_(*p <* 0.01). This correlation is still statistically significant after adjusting for multiple comparisons using Bonferroni correction (*p <* 0.05).
- Increases in the F10.7 index are associated with decreases in the PGA, i.e. positive *ΔF*10.7_*t*_ are associated with negative (*p <* 0.01). Furthermore, increases in the F10.7 index anticipate decreases in the PGA, i.e. positive *ΔF*10.7_*t* − 1_ are associated with negative (*p <* 0.01). Both correlations are still statistically significant after adjusting for multiple comparisons using Bonferroni correction (*p <* 0.05).

**Fig. 6:**
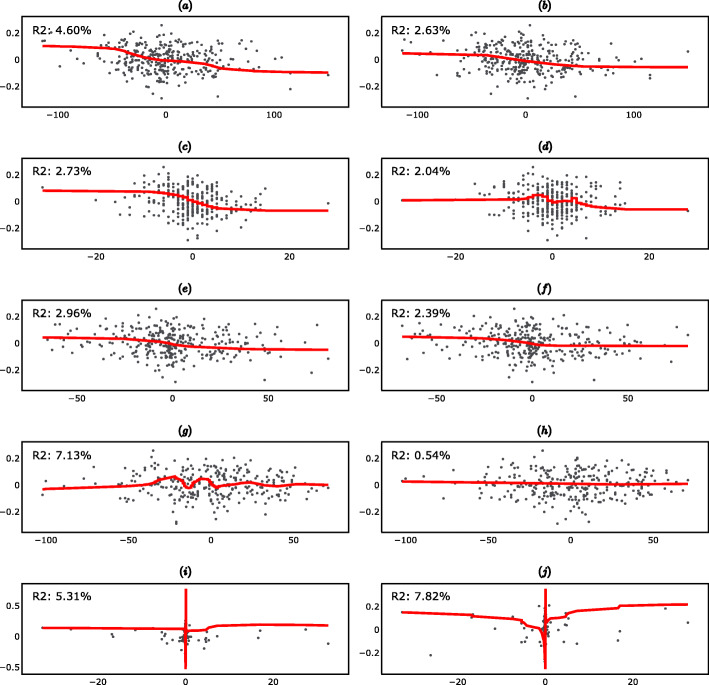
Long-Term Kernel Regressions (297-day differences) **a** Scatter plot of ΔPGA_t_ vs ΔR_t_. **b** Scatter plot of ΔPGA_t_ vs ΔR_t-1_. **c** Scatter plot of ΔPGA_t_ vs ΔAP_t_. **d** Scatter plot of ΔPGA_t_ vs ΔAP_t-1_. **e** Scatter plot of ΔPGA_t_ vs ΔF10.7_t_. **f** Scatter plot of ΔPGA_t_ vs ΔF10.7_t-1_. **g** Scatter plot of ΔPGA_t_ vs ΔAU_t_. **h** Scatter plot of ΔPGA_t_vs ΔAU_t-1_. **i** Scatter plot of ΔPGA_t_ vs ΔPF_t_. **j** Scatter plot of ΔPGA_t_ vs ΔPF_t-1_.

Figure 6 (supplementary section) shows the fitted Kernel regressions. The interpretation of the R-squared is the same as in Section 2.3.

**Fig. 7:**
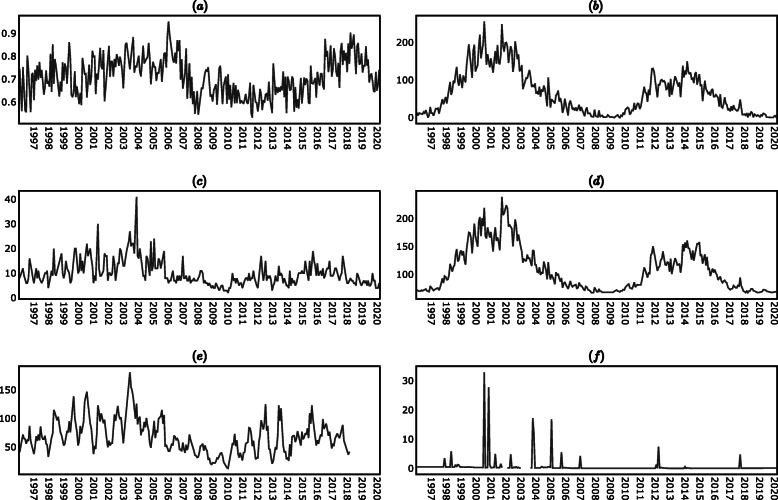
Levels time series. **a** PGA_t_. **b** R_t_. **c** AP_t_. **d** F10.7_t_ **e** AU_t_. **f** PF_t_

**Fig. 8:**
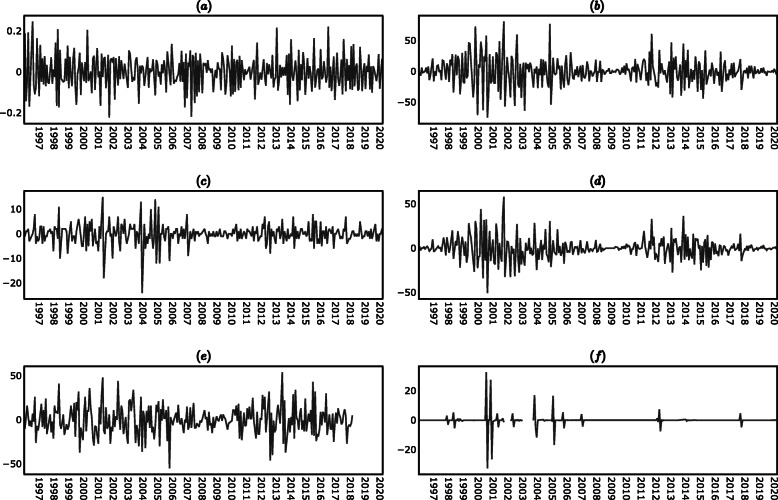
27-day differences time series. **a** PGA_t_. **b** R_t_. **c** AP_t_. **d** F10.7_t_ **e** AU_t_. **f** PF_t_

**Fig. 9:**
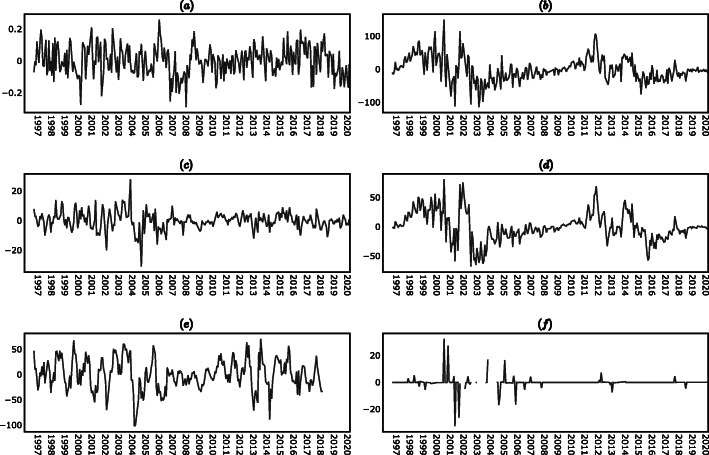
297-day differences time series. **a** PGA_t_. **b** R_t_. **c** AP_t_. **d** F10.7_t_ **e** AU_t_. **f** PF_t_

## Discussion

Overall, this study demonstrates the presence of a seasonality cycle of 16 years as an important factor underlying the variance of the SLE global disease activity time series, and suggests an association of SLE global disease activity with geomagnetic activity, sunspot numbers, and high energy proton fluxes.

The time series decomposition of the 27-day PGA averages over a period of 24 years revealed that seasonality (one cycle every 16 years) explains a significant fraction of the variance of the time series. A previous study from the Hopkins Lupus Cohort showed no evidence of short-term (within 1 year) seasonality of global SLE activity expressed by PGA [10]. While many short-, mid-, and long-term periodicities in global climate, solar and geomagnetic activity have been described [20, 21], only the N-S (North-South) asymmetry periodicity which is 16.5 years for both small and large sunspots is similar to the periodicity described in our PGA time series. There are a growing number of examples where the immune system undergoes complex temporal oscillations, some well-known such as periodic fevers during malarial infection, familial Mediterranean fever, cyclic neutropenia, and other less known examples including oscillations in the levels of NFκB [22], HeS1 [23], and the tumor suppressor protein p53 [24]. Apart from these, the immunological literature includes a variety of isolated reports of oscillations [25]. Overall, however, there is still relatively little understanding of the causes and implications of oscillatory behavior of the immune system and its clinical implications.

Oscillatory behavior is an inherent characteristic of solar output and thus of geomagnetic activity. Yearly sunspot number analysis reveals periodicities, (in order of decreasing power) as 11-years, 38.6-years, 22.7-years [20], but analysis of their double-peaked behavior demonstrated dominant periods of 9, 12, and 16.5 years [26]. In geomagnetic data, the periodicities (in order of decreasing power) are 11-years, 5.3-years, 30-years and 46.5-years and some others [20]. The majority of great geomagnetic storms take place in the descending phase of the solar cycles [27]. When a solar cycle is strong, the great geomagnetic storms concentrate during the period from the 2 years before the solar cycle peak time to the 3 years after the solar cycle peak time [27]. In a database [4] of 129,205 deaths from myocardial infarction recorded in Minnesota from 1968 to 1996, the yearly incidences of myocardial infarction were categorized by solar cycle stage: maximum, descending stage, minimum, and ascending stage. An excess of 220 deaths from myocardial infarction per year was seen during years of maximal vs. minimal solar activity [4]. On the other hand, during periods of solar minima the penetration of cosmic rays in Earth’s atmosphere is higher which results in atmospheric air ionization, which has been associated with atmospheric electricity, cloudiness, and climate, all affecting human health [28]. For example, in a populational retrospective study from Sao Paulo, strong correlations between cosmic ray exposure and total mortality, infectious diseases, maternal mortality, and perinatal mortality rates were observed [29].

We show that short-term (27-day) increases in geomagnetic activity Ap index (*p <* 0.1) and high energy proton fluxes (> 60 Mev) (*p <* 0.05) were associated with decreases in SLE disease activity, while short-term increases in the sunspot number index R anticipated decreases in the SLE disease activity expressed as PGA (*p <* 0.05). The long-term (297 day) relationships showed a stronger negative correlations between changes in the PGA and changes in the sunspot number index R (*p <* 0.01), AP index (*p* < 0.01), and the F10.7 index (*p* < 0.01), which remained statistically significant after adjusting for multiple comparisons using Bonferroni correction.

We can only speculate on potential underlying mechanisms. The combined effects of sunspot-induced changes in solar irradiance and increases in atmospheric greenhouse gases have been shown to offer the best explanation for the rise in average global temperature over the last century [30], which is also a tempting hypotheses for its effect on SLE disease activity, as temperature changes have been associated with SLE organ specific flares [11].

Increased geomagnetic disturbances are likely to activate the photo/magneto-reception system, including the ferromagnetic receptors and the cryptochrome protein [31–33], affect cell membrane excitability by the enhancement of ionic motion, signaling, and accumulation in ion channels, with primary focus on intracellular calcium [34], and ultimately regulate melatonin secretion and 24-h circadian rhythm disruption [35]. These effects have been postulated to underlie the association of geomagnetic disturbances with influenza pandemics [36]. Another hypothesis includes the possibility that diurnal geomagnetic variation is a zeitgeber for biological circadian rhythms, and animals could perceive geomagnetic storms as a disruption of the usual circadian rhythm [37].Whether these mechanisms could affect SLE disease incidence or disease activity remains unknown, but warrants further study.

The shortcoming of our study is the inclusion of data from only one geographical area. The short time span of the data, covering only 2–3 solar cycles, may have contributed to the loss of statistical power. Future studies should analyze SLE data covering a longer interval from many locations and organize them in geomagnetic coordinates [38].

## Supplementary Information

**Additional file 1. Supplementary section** The Ap index is a measure of the general level of geomagnetic activity over the globe for a given day. It is derived from measurements made at a number of stations world-wide of the variation of the geomagnetic field due to currents flowing in the earth's ionosphere and, to a lesser extent, in the earth's magnetosphere. The official values for Ap are calculated by the GeoForschungsZentrum Helmholtz Centre Potsdam (Germany) [39]. The sunspot number index R is a measure of the area of solar surface covered by spots. As the number of spots increases and their magnetic complexity grows, they become likely sources of large eruptive energy releases known as solar flares. The sunspot number index is also often called Wolf number in reference to the Swiss astronomer J. R. Wolf who introduced this index in 1848 [40]. The sun emits radio energy with slowly varying intensity. This radio flux, which originates from atmospheric layers high in the sun's chromosphere and low in its corona, changes gradually from day to day in response to the number of spot groups on the disk. Solar flux from the entire solar disk at a frequency of 2800 MHz has been recorded routinely by a radio telescope near Ottawa since February 1947 and is called the F10.7 index^40^. The AU (amplitude upper) index describes the disturbance level recorded by auroral zone magnetometers [40]. The radiation hazard from solar proton events (SPEs) has been characterized in terms of integral fluxes above selected threshold energies. We used high energy proton fluxes >60 MeV for our analysis [41].

## Data Availability

The datasets during and/or analyzed during the current study available from the corresponding author on reasonable request.
