# Supplementary material for: Systemic lupus Erythematosus and geomagnetic disturbances: a time series analysis
Source: Environ Health. 2021 Mar 16;20:28. doi: 10.1186/s12940-021-00692-4 (PMC7962208; doi:10.1186/s12940-021-00692-4)
Supplement: Supplementary file 1 — Additional file 1. Supplementary section The Ap index is a measure of the general level of geomagnetic activity over the globe for a given day. It is derived from measurements made at a number of stations world-wide of the variation of the geomagnetic field due to currents flowing in the earth's ionosphere and, to a lesser extent, in the earth's magnetosphere. The official values for Ap are calculated by the GeoForschungsZentrum Helmholtz Centre Potsdam (Germany) [39]. The sunspot number index R is a measure of the area of solar surface covered by spots. As the number of spots increases and their magnetic complexity grows, they become likely sources of large eruptive energy releases known as solar flares. The sunspot number index is also often called Wolf number in reference to the Swiss astronomer J. R. Wolf who introduced this index in 1848 [40]. The sun emits radio energy with slowly varying intensity. This radio flux, which originates from atmospheric layers high in the sun's chromosphere and low in its corona, changes gradually from day to day in response to the number of spot groups on the disk. Solar flux from the entire solar disk at a frequency of 2800 MHz has been recorded routinely by a radio telescope near Ottawa since February 1947 and is called the F10.7 index40. The AU (amplitude upper) index describes the disturbance level recorded by auroral zone magnetometers [40]. The radiation hazard from solar proton events (SPEs) has been characterized in terms of integral fluxes above selected threshold energies. We used high energy proton fluxes >60 MeV for our analysis [41]. [file 12940_2021_692_MOESM1_ESM.docx]

**SUPPLEMENTARY SECTION**

The Ap index is a measure of the general level of geomagnetic activity over the globe for a given day. It is derived from measurements made at a number of stations world-wide of the variation of the geomagnetic field due to currents flowing in the earth's ionosphere and, to a lesser extent, in the earth's magnetosphere. The official values for Ap are calculated by the GeoForschungsZentrum Helmholtz Centre Potsdam (Germany)^39^.

The sunspot number index R is a measure of the area of solar surface covered by spots. As the number of spots increases and their magnetic complexity grows, they become likely sources of large eruptive energy releases known as solar flares. The sunspot number index is also often called Wolf number in reference to the Swiss astronomer J. R. Wolf who introduced this index in 1848^40^.

The sun emits radio energy with slowly varying intensity. This radio flux, which originates from atmospheric layers high in the sun's chromosphere and low in its corona, changes gradually from day to day in response to the number of spot groups on the disk. Solar flux from the entire solar disk at a frequency of 2800 MHz has been recorded routinely by a radio telescope near Ottawa since February 1947 and is called the F10.7 index^40^.

The AU (amplitude upper) index describes the disturbance level recorded by auroral zone magnetometers^40^.

The radiation hazard from solar proton events (SPEs) has been characterized in terms of integral fluxes above selected threshold energies. We used high energy proton fluxes >60 MeV for our analysis^41^.

Figure 2: 27-day PGA average time series analysis.


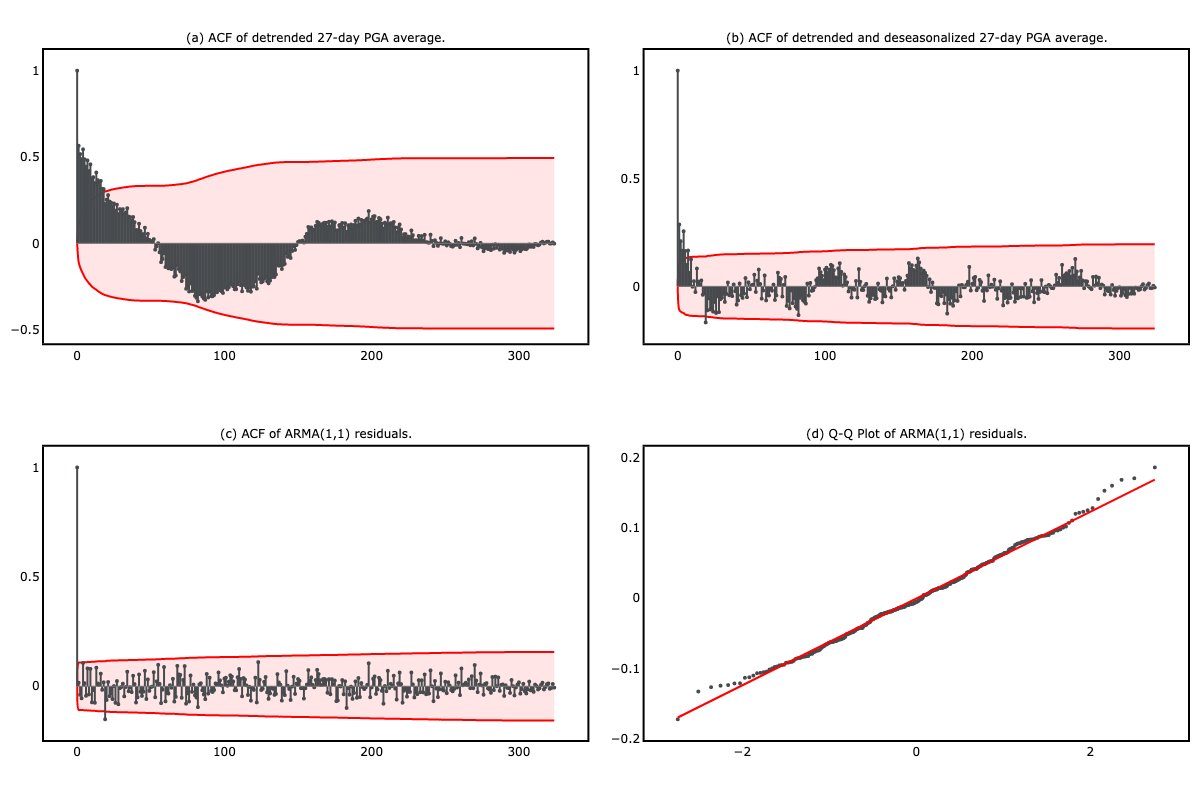


Figure 3: Short-Term Linear Regressions (27-day differences).


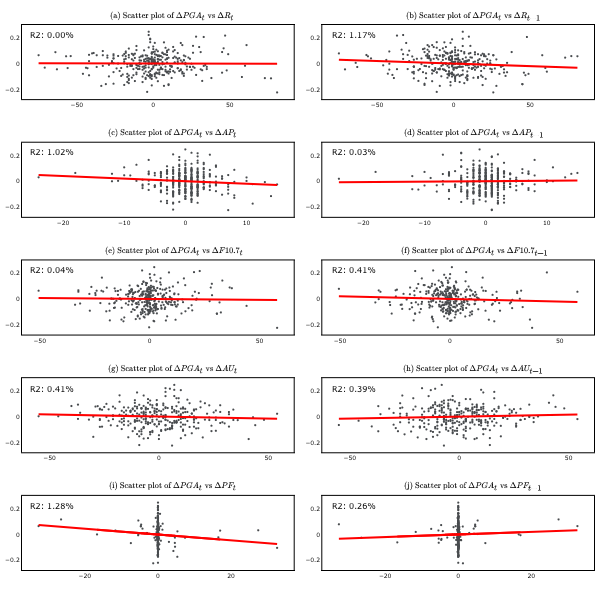


Figure 4: Long-Term Linear Regressions (297 day differences).


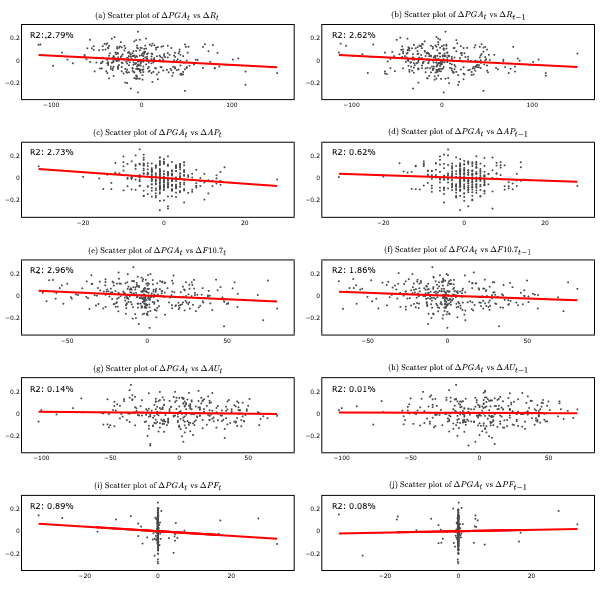


Figure 5: Short-Term Kernel Regressions (27-day differences).


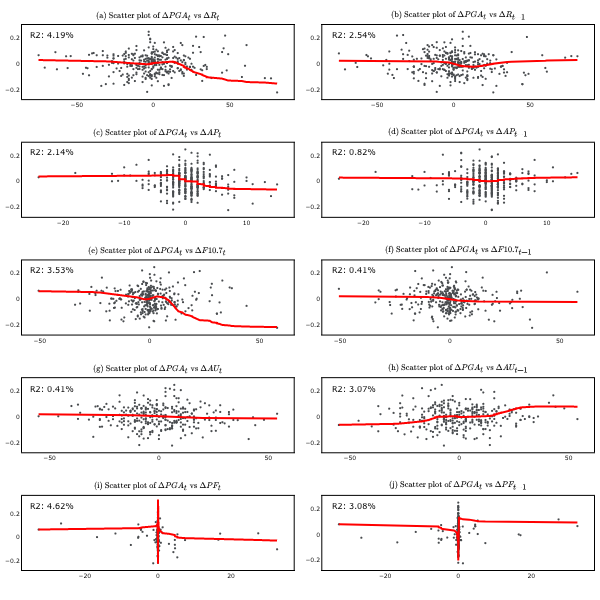


Figure 6: Long-Term Kernel Regressions (297-day differences).

**
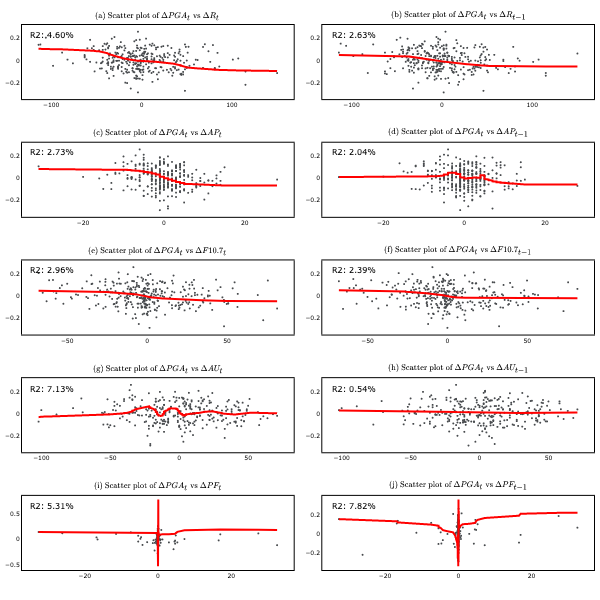
**

Figure 7: Levels time series.

Figure 8: 27-day differences time series.


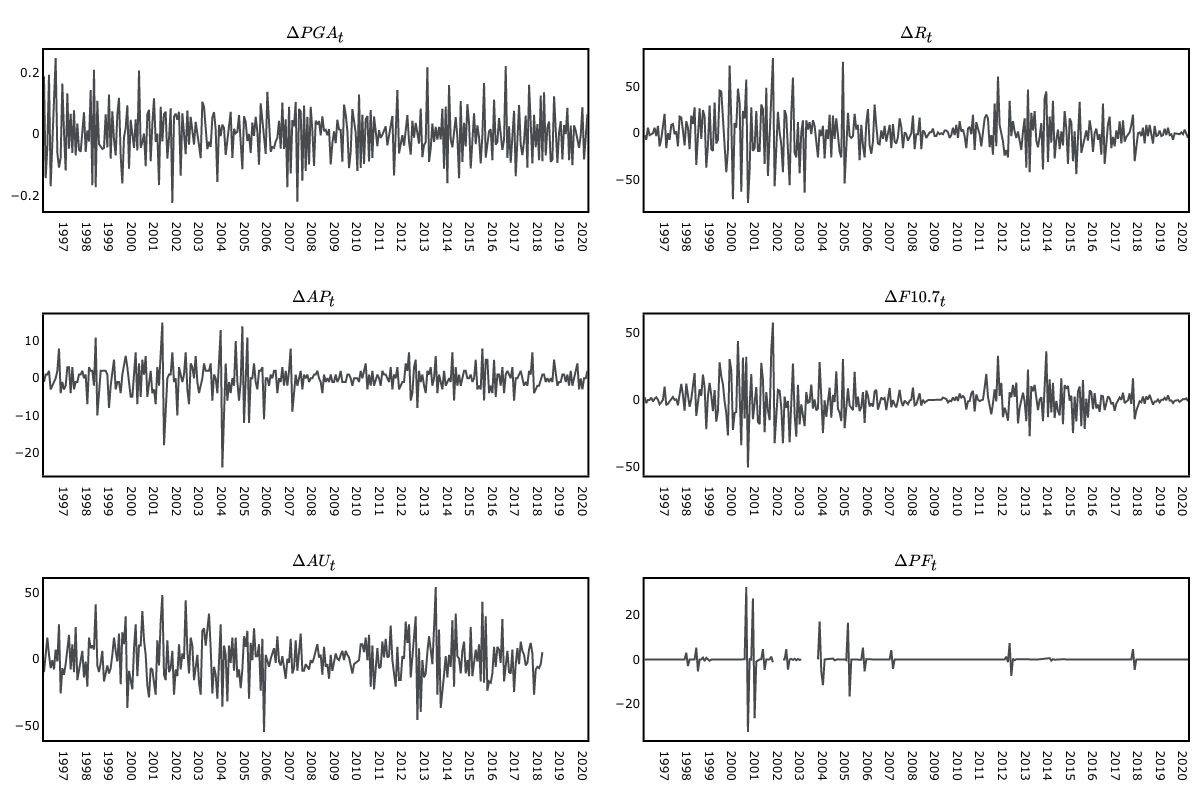


Figure 9: 297-day differences time series.


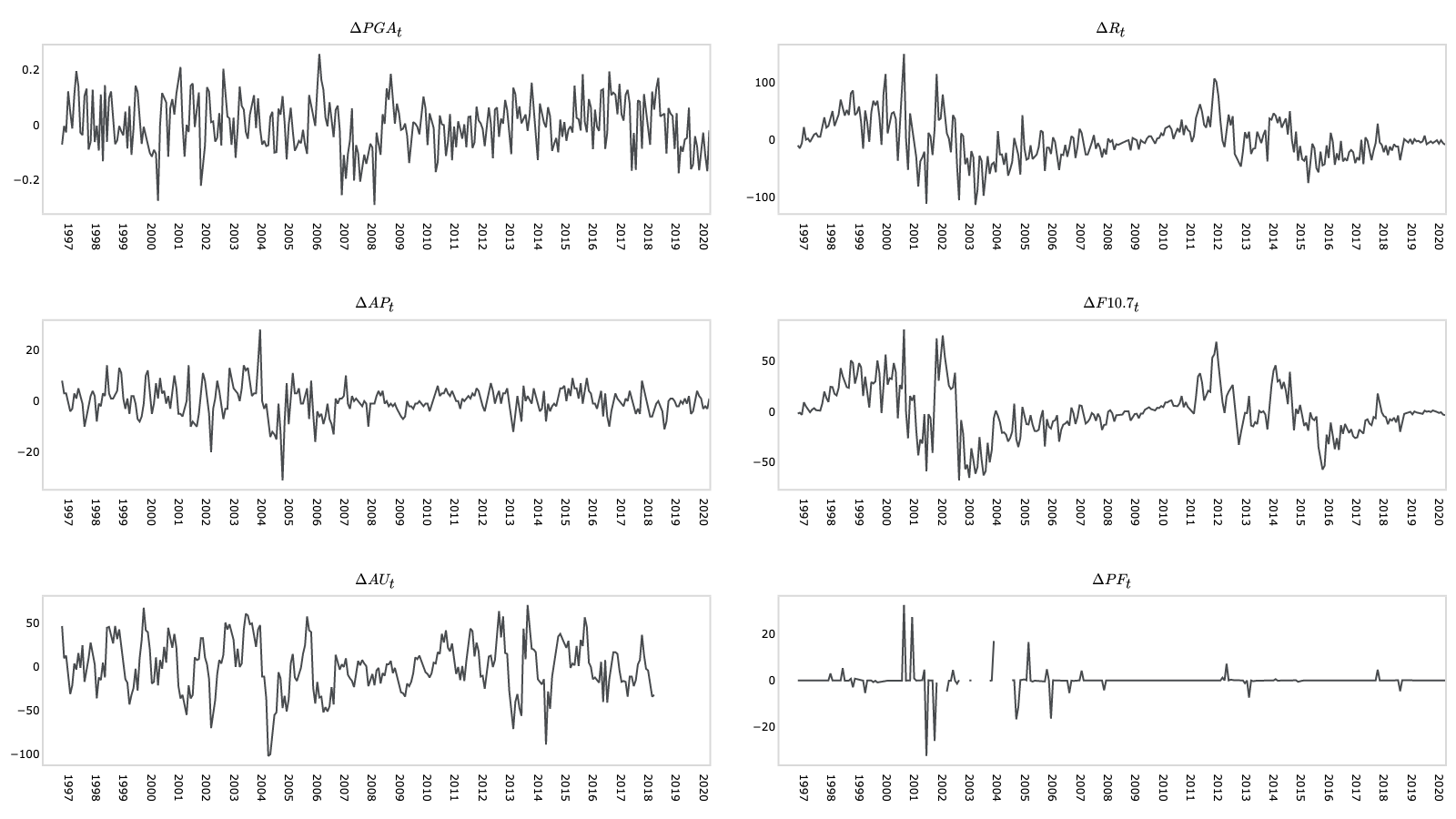

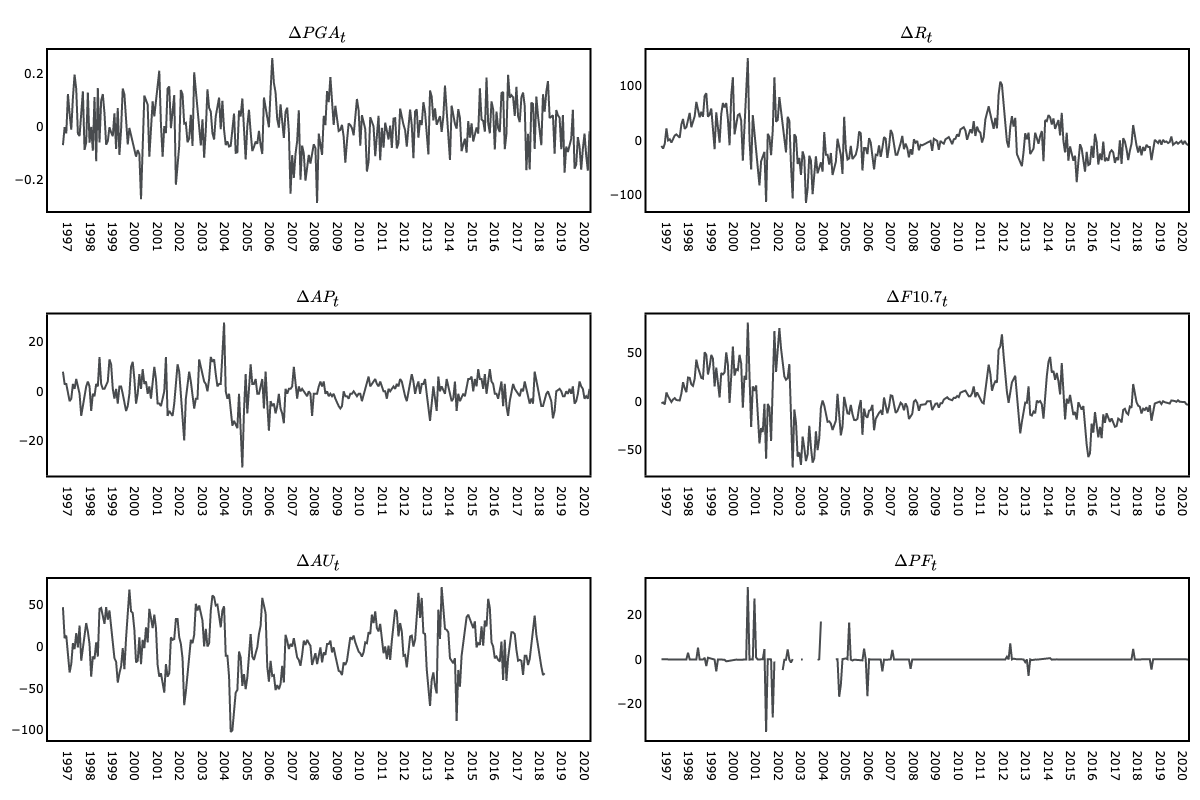


Table 6: Descriptive statistics of levels time series.

|  | PGA | R | AP | F10.7 | AU | PF |
| --- | --- | --- | --- | --- | --- | --- |
| Count | 327 | 327 | 327 | 327 | 300 | 315 |
| Mean | 0.71 | 64.92 | 10.08 | 107.64 | 68.57 | 0.68 |
| Standard Deviation | 0.08 | 57.80 | 5.04 | 38.92 | 29.09 | 2.92 |
| Minimum | 0.53 | 0.00 | 2.00 | 67.00 | 13.00 | 0.05 |
| 25^th^ Percentile | 0.65 | 13.00 | 7.00 | 74.00 | 48.75 | 0.07 |
| 50^th^ Percentile | 0.71 | 53.00 | 9.00 | 96.30 | 65.00 | 0.12 |
| 75^th^ Percentile | 0.77 | 101.50 | 12.00 | 132.55 | 85.25 | 0.34 |
| Maximum | 0.95 | 256.00 | 41.00 | 238.90 | 181.00 | 32.93 |

Table 7: Descriptive statistics of 27-day differences time series.

|  | PGA | R | AP | F10.7 | AU | PF |
| --- | --- | --- | --- | --- | --- | --- |
| Count | 327 | 327 | 327 | 327 | 300 | 315 |
| Mean | 0.001 | -0.046 | -0.009 | -0.002 | -0.030 | -0.001 |
| Standard Deviation | 0.079 | 21.635 | 4.040 | 12.147 | 16.048 | 4.007 |
| Minimum | -0.223 | -75.000 | -24.000 | -50.500 | -55.000 | -32.660 |
| 25^th^ Percentile | -0.049 | -11.000 | -2.000 | -5.150 | -10.000 | -0.010 |
| 50^th^ Percentile | 0.001 | 0.000 | 0.000 | -0.200 | -1.000 | 0.000 |
| 75^th^ Percentile | 0.056 | 10.000 | 2.000 | 4.000 | 10.000 | 0.010 |
| Maximum | 0.246 | 81.000 | 15.000 | 58.000 | 54.000 | 32.600 |

Table 8: Descriptive statistics of 297-day differences time series.

|  | PGA | R | AP | F10.7 | AU | PF |
| --- | --- | --- | --- | --- | --- | --- |
| Count | 318 | 318 | 318 | 318 | 291 | 294 |
| Mean | 0.002 | -0.362 | -0.085 | -0.081 | 0.107 | -0.038 |
| Standard Deviation | 0.092 | 36.523 | 5.848 | 24.539 | 29.845 | 4.213 |
| Minimum | -0.289 | -114.000 | -31.000 | -67.900 | -102.000 | -32.620 |
| 25^th^ Percentile | -0.068 | -23.000 | -3.000 | -12.550 | -17.000 | -0.030 |
| 50^th^ Percentile | 0.002 | -3.000 | 0.000 | -1.350 | 1.000 | 0.000 |
| 75^th^ Percentile | 0.064 | 18.750 | 3.000 | 11.050 | 20.000 | 0.010 |
| Maximum | 0.259 | 150.000 | 28.000 | 81.300 | 71.000 | 32.540 |

Table 9: KPSS test p-values.

|  | Levels time series | 27-day differences time series | 297-day differences time series |
| --- | --- | --- | --- |
| $\mathrm{PGA}$PGA | >0.10 | >0.10 | >0.10 |
| R | <0.05 | >0.10 | >0.10 |
| AP | <0.01 | >0.10 | >0.10 |
| F10.7 | <0.05 | >0.10 | >0.10 |
| AU | <0.05 | >0.10 | >0.10 |
| PF | <0.01 | >0.10 | >0.10 |
